# A Review of the Health Benefits of Cherries

**DOI:** 10.3390/nu10030368

**Published:** 2018-03-17

**Authors:** Darshan S. Kelley, Yuriko Adkins, Kevin D. Laugero

**Affiliations:** 1 US Department of Agriculture, Agricultural Research Service, Western Human Nutrition Research Center, Davis, CA 95616, USA; yuriko.adkins@ars.usda.gov (Y.A.); kevin.laugero@ars.usda.gov (K.D.L.); 2 Department of Nutrition, University of California, Davis, CV 95616, USA

**Keywords:** cherries, polyphenols, anthocyanins, inflammation, oxidative stress, chronic diseases

## Abstract

Increased oxidative stress contributes to development and progression of several human chronic inflammatory diseases. Cherries are a rich source of polyphenols and vitamin C which have anti-oxidant and anti-inflammatory properties. Our aim is to summarize results from human studies regarding health benefits of both sweet and tart cherries, including products made from them (juice, powder, concentrate, capsules); all referred to as cherries here. We found 29 (tart 20, sweet 7, unspecified 2) published human studies which examined health benefits of consuming cherries. Most of these studies were less than 2 weeks of duration (range 5 h to 3 months) and served the equivalent of 45 to 270 cherries/day (anthocyanins 55–720 mg/day) in single or split doses. Two-thirds of these studies were randomized and placebo controlled. Consumption of cherries decreased markers for oxidative stress in 8/10 studies; inflammation in 11/16; exercise-induced muscle soreness and loss of strength in 8/9; blood pressure in 5/7; arthritis in 5/5, and improved sleep in 4/4. Cherries also decreased hemoglobin A1C (HbA1C), Very-low-density lipoprotein (VLDL) and triglycerides/high-density lipoprotein (TG/HDL) in diabetic women, and VLDL and TG/HDL in obese participants. These results suggest that consumption of sweet or tart cherries can promote health by preventing or decreasing oxidative stress and inflammation.

## 1. Introduction

Epidemiological studies indicate an inverse association among fruit and vegetable intake and the risk for several chronic inflammatory diseases [1,2]. Consumption of fruits and vegetables has been reported to reduce the risks of all-cause mortality, and morbidity and mortality from cardiovascular disease (CVD), stroke, diabetes, and some cancers [3,4,5,6]. Besides providing essential vitamins, minerals, carotenoids and dietary fiber, fruits contain polyphenols [7,8,9] which are believed to decrease risk for metabolic syndrome, diabetes, nonalcoholic fatty liver disease (NAFLD) and CVD [10,11,12,13,14,15,16,17].

The cherry fruit is a nutrient dense food with relatively low caloric content and significant amounts of important nutrients and bioactive food components including fiber, polyphenols, carotenoids, vitamin C, and potassium [18]. In addition, cherries are also good source of tryptophan, serotonin, and melatonin [19,20]. While there are more than a hundred cultivars of cherries, they are grouped into two major types, the sweet (*Prunus avium* L.) and tart (*Prunus cerasus* L.) cherries [21]. The most commonly grown cultivar of sweet cherries in the USA is Bing and for the tart is Montmorency. The majority of sweet cherries are consumed fresh with the remaining 20–25% processed as brined, canned, frozen, dried, or juiced. In contrast, 97% of tart cherries are processed primarily for cooking and baking [18].

Both sweet and tart cherries are rich in polyphenols [18,21,22]. Many factors including the cultivar, stage of ripening, portion of fruit, storage, and others contribute to the polyphenolic concentration and composition of cherries [22]. Cyanidin-3-glucoside and cyanidine-3-rutinoside are the major anthocyanins in both Bing and Montmorency cherries. In addition to the anthocyanins, cherries are also rich in hydroxycinnamates and Flavin-3-ols. Hydroxycinnamates and Flavin-3-ols respectively make up about 25% and 40% of the total phenolics in Montmorency cherries and 50% and 5% in Bing cherries [23,24]. Other flavonoids make up the remainder of the phenolics in both sweet and tart cherries [18].

Published literature suggested that tart cherries had higher concentrations of total phenolic compounds while the sweet cherries contained more anthocyanins [18]. Thus, the total phenolics for the flesh, pits, and skins of Bing cherries were 134, 92, 333 mg/100 g fresh weight, and the corresponding values for Montmorency cherries were 301, 157, and 558 mg/100 g, respectively [18,25]. The total anthocyanin concentrations for Bing cherries were 26.0, 10.4, and 60.6 mg/100 g of flesh, pits, and skins, while the corresponding values for the Montmorency cherries were 0.0, 0.8, and 36.5 mg/100 g, respectively [18,26]. Anthocyanin concentrations in 10 other cultivars of red sweet cherries (Benton, Black Gold, Glacier, Hedelfingen, Kiona, Kordia, Kristin, Regina, Selah and Skeena) ranged from 82–297 mg/100 g; yellow sweet cherries (Gold and Rainer) 2–41 mg/100 g, and for red sour cherries (Montmorency and Balton) 27–76 mg/100 g [27]. Total anthocyanin in six other cultivars of sweet cherries (Delta Marca, Celste, Bigarreau, Durone Nero, Lapins and Moretta) ranged from 2.1–344.9 mg/100 g fresh weight (approximately 0.6–22% of the total phenolics) [28]. Thus, there is wide range in the concentration of anthocyanins in the different cultivars of cherries which may be due to the factors listed above and the precision of the analytical methods used. Further analyses under identical conditions are needed to compare the phenolic composition of specific cultivars of cherries. Melatonin is another antioxidant which is linked to sleep regulation, and is found in both sweet and tart cherries; its concentration ranged from 10–20 ng/g fresh weight in both Hongdeng and Rainier ripe sweet cherries [29] and it was 2.1 ng/g and 13.5 ng/g, in Balton and Montmorency tart cherries, respectively [30].

The antioxidant capacity of sweet and tart cherries varied when compared using different test systems. Thus, in the oxygen radical absorbing capacity (ORAC) and ferric reducing ability of plasma (FRAP) assays, the edible portion of the Montmorency cherries had greater antioxidant activity than those in the sweet cherries [18], however, in a liposome-based system, the sweet cherries exhibited the highest antioxidant activity [27]. Recent in vitro studies have shown that the antioxidant effect between anthocyanins and quercetins/ascorbic acid was synergistic in the Sandra Tardiva cultivar of sweet cherries [22].

Both anthocyanins and hydoxycinnamates are believed to be rapidly absorbed in humans reaching maximum plasma concentrations in less than 2 h and are quickly eliminated [31,32]. Low plasma concentrations may also result from the inability to measure some of their metabolites. Results of a recent study in healthy men using ^13^C labelled cyanidine-3-glucoside, reported that the serum peak concentration reached at 10.2 h after the ingestion and metabolites of anthocyanin were present in the serum for greater than 48 h. The amount of ^13^C in urine, fecal and breath samples collected in 48 h accounted for 12.3% of the ^13^C consumed [33]. These findings suggest that anthocyanins have a minimum of 12.3% bioavailability and their metabolites remain in circulation longer than previously believed. Further studies are needed to confirm the bioavailability of anthocyanins.

Given the high concentrations of bioactive compounds (e.g., anthocyanins, hydoxycinnamates, Flavin-3-ols) in cherries, it is not surprising that cherry consumption promotes health. Results from published animal and human studies suggest that consumption of cherries may reduce the risk of several chronic inflammatory diseases including, arthritis, cardiovascular disease (CVD), diabetes, and cancer. Furthermore, there is evidence that cherry consumption may improve sleep, cognitive function, and recovery from pain after strenuous exercise. Some of these findings have been reviewed [18,34,35]. Since the last published review on the health benefits of cherries (Bell et al., 2014), another review has been recently accepted for publication [36]. This recent review focuses on the anti-arthritic effects of tart cherries and the fate of phytochemicals in the human gastrointestinal tract. Our review that follows includes all published effects of both sweet and tart cherries on markers of oxidative stress, inflammation, exercise-related muscle damage, arthritis, diabetes, cardiovascular disease, sleep, and cognitive functions. Our objective is to summarize the results from human studies regarding the health benefits of cherries or products (juice, powder, concentrate, capsules) made from sweet or tart cherries. Results from animal and cell culture studies are also included to support the findings from human studies or to highlight the potential mechanisms involved. We used PubMed and Google scholar to find published human studies with cherries or cherry products. A total of 29 studies were found and are discussed below.

## 2. Clinical Studies Involving Consumption of Cherries and Their Products

We found a total of 29 human studies that examined health promoting effects of cherries or products derived from cherries. Twenty of these studies used tart cherries or products, 7 used sweet cherries or products, and 2 used fresh and canned cherries but did not specify whether the products were derived from tart or sweet cherries. Since cherries used as fresh are often sweet, it is likely that these two studies used either sweet or both sweet and tart cherries. All published human studies with cherries were grouped according to the clinical end points being investigated and are listed in Table 1. Responses tested include oxidative stress (10 studies); markers of inflammation (16 studies); exercise induced pain, muscle damage and recovery (9 studies); risk factors for diabetes and CVD including hemoglobin A1C (HbA1C), blood pressure and lipids (9 studies); markers for arthritis besides inflammation (5 studies); quality and quantity of sleep (4 studies); stress, anxiety, mood, memory and cognitive functions (3 studies). Many of the studies tested more than one type of those response variables.

Table 2 lists characteristics of the study participants, study design and duration, treatment and dose, and the major findings of the individual studies. Study participants included ranged from young athletes to elderly with dementia, insomnia, arthritis, or other chronic conditions. Both male and female subjects were included with a sample size ranging from 9 to 633. The daily dose of cherries used ranged from 45 to 270 cherries (anthocyanins 55–720 mg/day), which were served as a single dose or split into 2 or 3 doses. Nineteen of the studies used randomized, placebo-controlled design with a cross-over or parallel format; there were 10 studies which did not include the control groups and tested the responses before and after cherry consumption. Study duration ranged from 5 h to 3 months. Results from these studies are discussed below.

### 2.1. Effects of Cherries on Oxidative Status, Inflammatory Response, Exercise-Induced Pain, Muscle Damage, and Recovery

Polyphenols, melatonin, carotenoids, and vitamins E and C all contribute to the antioxidant and anti-inflammatory properties of cherries [18,21,22,34,67]. Markers of oxidative stress monitored in the human studies with cherries included plasma/serum ORAC, FRAP, trolox equivalent antioxidant capacity (TEAC), F-2 isoprostane, nitrotyrosine (NT), superoxide dismutase (SOD), lipid peroxidation (LOOH), total serum antioxidant status (TAS), thiobarbituric acid (TBARS) and urinary isoprostanes; ex vivo oxidation of 2,2-diphenyl-1-picrylhydrazyl (DPPH). Inflammation was assessed by examining plasma concentrations of C-reactive protein (CRP), erythrocyte sedimentation rate (ESR), nitric oxide (NO), cytokines (IL-1, IL-6, IL-8, TNF α, MCP-1, IL-1 receptor antagonist). We note that the biological significance of the in vitro measures of oxidative stress (ORAC, FRAP, TAS) remains debatable. Muscle damage was evaluated by determining serum concentrations of creatine kinase (CK) and lactate dehydrogenase (LDH), recovery was estimated by the restoration of strength and decrease in muscle soreness, and muscle pain levels were determined by the visual analog scale (VAS).

#### 2.1.1. Antioxidant Effects of Consuming Cherries

Out of a total of 29 published human studies, 10 monitored the effects of cherries and cherry products on markers of oxidative stress (Table 1 and Table 2). Oxidative stress was decreased (or antioxidant capacity increased) in 8 studies [37,38,39,40,41,42,43,48], and it did not change in 2 studies [46,47]. Markers of antioxidant capacity that were altered by cherry consumption included increased plasma ORAC [40], FRAP [42], serum TAS [37,39,41], decreased plasma F2-isoprostane [43] and LOOH [44], and increased urinary antioxidant capacity [38]. The lack of an effect of cherry juice on oxidative stress in the study by [47] may have been due to the type of the exercise examined (water polo) which did not increase oxidative stress. The nature of the supplements (tart cherry powder capsules) or the short-term supplementation around a single bout of resistance training may be the reason for the lack of an effect of tart cherry powder on oxidative stress in the study by [46] those studies showing antioxidant effects included both sweet and sour cherries. Taken together, these findings from human studies suggest that both sweet and tart cherries reduce oxidative stress. This inference is also supported by the results from animal and cell culture studies in which cherry extracts increased the hepatic activity of antioxidant enzymes in liver and decreased the iron or copper induced lipid peroxidation in vitro [21,36].

#### 2.1.2. Cherry Intake and Inflammation

Sixteen human studies investigated the effects of consuming cherries or cherry products on markers of inflammation which were shown to be decreased in 11 studies [23,37,39,40,41,44,46,48,49,50,51,52] did not change in 4 studies [47,54,55,56], and increased in 1 study [53] (Table 1). Markers of inflammation that were decreased included ESR [52] plasma concentrations CRP [23,39,44,48,50,55], TNF α [41,46,51,68], IL-6 [39,41,44,46,49], IL-8 [39,41,44,46,49], RANTES [23], NO [23], MCP-1 [52], and upper respiratory tract symptoms [50]. Plasma CRP was also decreased by approximately 25% within 5 h of a bolus of 45 fresh Bing cherries compared with baseline values, although it did not attain significance [40]. In the two studies by Levers et al. [41,46], the pre-exercise plasma levels of inflammatory cytokines (IL-6, IL-8, and TNF α did not differ between the placebo and tart cherry groups, but their post exercise plasma concentrations were significantly lower in the cherry group. In the study by Kelley et al. [51] plasma concentrations of other inflammatory markers were also altered by consumption of sweet cherries, including decreases in IL-18 and ferritin, and an increase in IL-1R antagonist.

In contrast, no change in serum CRP and IL-6 resulted from consumption of sweet cherry juice for 6 and 12 weeks in elderly subjects with dementia (mean age 80 years), [54]. Besides the age of the study participants, the low dose (138 mg/day) of anthocyanins used in this study may be the reason for the lack of an effect of cherry juice on serum markers of inflammation. In the athletes participating in a water polo game, consumption of tart cherry juice had no effect on plasma CRP and IL-6, which may be because the non-weight bearing sport did not increase inflammation [47]. In another study, with obese subjects, consumption of fresh sweet cherries for 4 weeks did not alter urinary prostaglandin E2 and Thromboxane B2, serum CRP, and homocysteine when compared to the baseline [56]. The failure to detect changes in those markers, in this study, may be due to the variations in the anthocyanin concentrations of different batches of fresh sweet cherries used, which varied almost 20-fold during intervention. In another study, unexpectedly, the serum levels of IL-1β, TNF α, and IL-8 were increased in the blood samples drawn at 1 a.m., following cherry drinks with dinner [53]. Increase in these markers of inflammation in this study correlated with serum concentrations of 5-hydroxyindole acetic acid, a metabolite of melatonin. Other studies have shown that melatonin increased serum concentrations of IL-1 β and TNF α, both of which induce sleep [69]. Despite some inconsistences, the findings discussed above support the anti-inflammatory effects of cherries in humans. This conclusion is also supported by the inhibition of enzymes cyclooxygenase-1 and 2 by cherry extracts [27,70] and of nuclear factor-κB in cultured human blood monocytes by anthocyanins [71].

#### 2.1.3. Effects of Consuming Cherries on Exercise Induced Muscle Damage and Recovery

Exercise-induced muscle pain, soreness and loss of strength were significantly reduced by cherry consumption in 8 out of 9 studies [37,39,41,44,46,49,57,58], but were not different from the placebo in one study that involved water polo athletes [47]. Post-exercise muscle damage as determined by plasma concentration of CK and LDH when compared with placebo groups was reduced by cherry products in one [39], but not in other studies [37,44,49]. The attenuation of exercise-induced muscle damage by cherries seems to be related to the antioxidant and anti-inflammatory properties of anthocyanins and other phenolic compounds found in cherries [35]. All the exercise related studies were conducted with tart cherry products ranging from the equivalent of 50 to 270 cherries a day.

### 2.2. Effects of Consuming Cherries on Risk Factors for Diabetes and Cardiovascular Disease

#### 2.2.1. Cherry Intake and Diabetes

Supplementation with cherries or cherry products did not alter fasting or randomly sampled blood glucose and fasting insulin in healthy study participants [23,45]. In a study with diabetic women, concentrated tart cherry juice at 40 mL/day (anthocyanins 720 mg/day) for 6 weeks significantly decreased hemoglobin A1C (HbA1C) when compared with the levels before the supplementation; fasting blood glucose (FBG) was also decreased by 8% but did not attain significance [59]. Although this study did not include a control group, these findings are consistent with those found in animal and in vitro studies. Consumption of extracts from both sweet and tart cherries prevented alloxan-induced diabetes in rats [72] and in mice [73]. Adding cherry extract or purified anthocyanins to the high fat diets fed to mice and rats decreased circulating glucose, insulin and liver triglycerides when compared with those groups fed the high fat diets without cherry products [74,75,76]. Sweet cherry fractions rich in anthocyanins, hydroxycinnamic acid, or flavanols increased glucose consumption by cultured HepG2 cells [77]. Aqueous extracts prepared from several cultivars of sweet cherries inhibited the enzyme α glucosidase, which is involved in the intestinal absorption of carbohydrates [78]. Similarly, tart cherry juice and one of its main polyphenols known as chlorogenic acid inhibited enzymes α glucosidase and dipeptidyl peptidase-4 which are involved in promoting diabetes [79,80]. Tart cherry extract and select anthocyanins purified from it also inhibited the activity of human α amylase in vitro [81]. In vitro addition of anthocyanins (delphindin-3-glucoside and cyandin-3-galactoside) increased glucose-stimulated insulin secretion by cultured rodent pancreatic beta cells [82]. Results from human, animal, and cell culture studies suggest that anthocyanins may decrease blood glucose by slowing glucose production from complex carbohydrates, hepatic glucose output, decreasing the production of glucagon by pancreatic α cells, and increasing hepatic glucose uptake and production of insulin by pancreatic β cells [80]. Taken together, there exists evidence to suggest that cherry consumption may promote healthy glucose regulation. Future studies are needed to confirm whether these findings translate to reduced risk of diabetes.

#### 2.2.2. Cherry Intake and Blood Lipids

Consumption of sweet cherries or tart cherry concentrate by healthy adults did not alter concentrations of blood lipids, including triglycerides, low-density lipoprotein (LDL), very-low-density lipoprotein (VLDL), high-density lipoprotein (HDL), total cholesterol, number of different lipoprotein particles and their sizes in healthy adults [23,42]. In contrast to the studies with healthy participants, another study with overweight and obese subjects who had elevated blood lipids reported a decrease in VLDL and triglycerides/high-density lipoprotein (TG/HDL) ratio following consumption of tart cherry juice for 4 weeks [52]. It seems the lipid profile of study participants prior to the supplementation with tart cherries [52] versus sweet cherries [23] rather than the type of cherries may have contributed to the different results between these two studies. As stated above, cherry extracts and purified anthocyanins decreased liver triglycerides and cholesterol in mouse and rat models and prevented the high fat diet induced development of NAFLD [74,75,76].

#### 2.2.3. Cherry Intake and Blood Pressure

Effects of cherry consumption on blood pressure (BP) were examined in 7 studies; 3 of these studies examined the acute effects [60,61,62], and 4 examined the chronic effects of cherry consumption [42,51,59,62]. Both systolic blood pressure (SBP) and diastolic blood pressure (DBP) were significantly lowered within 2 h of a single dose of 300 mL of Bing cherry juice and returned to the baseline levels at 6 h in the young and elderly adults [62]. However, if the juice was served in 3 doses of 100 mL each at 0, 1, and 2 h there was no decrease in either SBP or DBP at 2 or 6 h These findings indicate that both the dose and time after ingestion are important in determining the BP lowering effects of cherry juice. Time dependent effects of tart cherry concentrate were also observed in two other studies where only the SBP was significantly decreased at 1 and 2 h after ingestion of Montmorency cherry concentrate, but not at 4 and 5 h after the supplementation [31,60]. The acute effects of cherry concentrate on BP were associated with increase in plasma concentrations of vanillic and protocatechuic acids, which are metabolites of cyanindin-3-glucoside [60].

In a study with diabetic women, 6-week supplementation with 40 g/day of tart cherry concentrate (anthocyanins 720 mg/day) significantly decreased both SBP and DBP when compared with the pre-supplementation values [59]. In another placebo controlled parallel study of elderly subjects 200 mL/day of Bing cherry juice (anthocyanins 138 mg/day) significantly decreased SBP, but not DBP at 6 and 12 weeks, when compared to the placebo group (Apple juice) [54]. Similarly, in another study with healthy adults, Bing cherries consumed at 280 g/day (anthocyanins 100 mg/day) for 28 days significantly decreased plasma concentration of endothelin-1 (ET-1) but the decrease in SBP did not attain significance [51]. In contrast to the above studies, supplementing at 30 mL/day tart cherry concentrate (anthocyanins 273 mg/day) for 6 weeks failed to decrease both SBP and DBP in healthy adults with relatively low mean SBP of 110, and DBP of 70 mm Hg [42]. Normal blood pressures of study participants, low dose of anthocyanins, and the time elapsed between consumption of cherry juice and the monitoring of blood pressure may have contributed to the lack of a decrease in BP in subjects consuming cherries. Further studies to determine the benefits of chronic consumption of cherries need to be conducted in participants with border line blood pressure.

The decrease in blood pressure caused by the prolonged consumption of cherries may have resulted from the decrease in endothelin-1 (ET-1) which is one of the most potent vasoconstrictors [51]. NO produced by endothelial NO synthase (eNOS) is an important vasodilator, and its expression was increased by the addition of cyanidin-3-glucosdie to cultured human umbilical vein endothelial cells and bovine vascular endothelial cells [83]. Hence, altered expression of both ET-1 and eNOS by cherry consumption may have contributed to the decrease in blood pressure.

Extracellular newly identified ligand for the receptor for advanced glycation end products (EN-RAGE) and plasminogen activator inhibitor-1 (PAI-1) are other risk factors for diabetes and CVD whose plasma concentrations were significantly decreased following the consumption of sweet cherries for 4 weeks by healthy study participants [51]. Plasma concentration of EN-RAGE was positively associated with concentrations of CRP, hemoglobin A1C, and fasting blood glucose [84]. PAI-1 is the major physiologic inhibitor of tissue-type plasminogen activator that prevents clot formation through fibrinolysis. Plasma concentration of PAI-1 correlates with metabolic syndrome and may predict future risk for type 2 diabetes mellitus (T2DM) and CVD [85]. Other in vitro studies demonstrated that anthocyanins inhibited expression of NF-κB, inflammatory cytokines, and adhesion molecules which are involved in the initiation and progression of CVD [86]. Adding tart cherry extract to the atherogenic diet fed to rabbits decreased plaque formation and improved cardiac functions [87]. Although further studies are needed, the available literature supports the conclusion that regular consumption of cherries may reduce the incidence of T2DM and CVD.

### 2.3. Effects of Consuming Cherries on Arthritis and Associated Risk Factors

The earliest study regarding the health benefits of fresh and canned cherries was conducted in 1950 in patients with gout [63]. Results from this study demonstrated that consumption of fresh or canned cherries prevented attacks of arthritis and restored the plasma uric acid (UA) concentrations to normal levels in all 12 patients. Furthermore, 4 patients reported greater freedom of joint movements in fingers and toes. These findings were published for more than 5 decades before the next human study regarding cherries and health was conducted by [40]. The study by Jacob et al. investigated the acute effects of ingesting a bolus of 45 sweet cherries in 10 young healthy women. They found that cherry consumption decreased plasma markers of oxidative stress and inflammation. Plasma UA concentration which is considered a marker for gout, was significantly reduced at 5 h after a dietary bolus of sweet cherries, but not at 1.5 and 3 h when compared to pre-challenge values. Results from recent studies regarding the effects of cherry consumption on plasma concentrations of UA have been variable. In one study, with obese subjects, consumption of tart cherry juice for 4 weeks significantly reduced plasma concentration of UA [52], while it was not altered by consumption of tart cherry juice within 6 weeks in patients with osteoarthritis [55], or within 7 days in water polo athletes [47]. Although the tart cherry juice did not decrease UA in patients with osteoarthritis, it significantly decreased plasma CRP and the Western Ontario McMaster Osteoarthritis Index. In a recent case-crossover study with 633 gout patients, consumption of fresh cherries or cherry extract over a 2-day period was associated with a 35% lower risk of gout attacks compared with no intake of cherries [64]. The effect of cherry intake persisted across subgroups stratified by sex, obesity status, purine intake, alcohol, diuretic, and antigout medications use. When cherry intake was combined with allopurinol use, the risk of gout attacks was 75% lower than during periods without either exposure. Anthocyanins inhibited the activity of Xanthine oxidase, the enzyme involved in UA synthesis, in vitro and also decreased serum UA concentration in hyperuricemic mice [88]. Similarly, tart cherry juice decreased the serum concentration of UA in hyperuricemic rats [89]. Although there are inconsistencies in the results from different human studies, taken together, findings support the conclusion that cherry consumption may reduce the incidence of arthritic attacks. These human findings regarding the reduction in arthritis by cherry consumption are consistent with the reduction of adjuvant- or collagen-induced arthritis in rat and mouse models by anthocyanins [5,90,91,92]. Suppression of the expression of NFκB, inflammatory cytokines, and inhibition of activities of enzymes cyclooxygenase-1 and -2 activities by purified anthocyanins and cherry extracts also supports the anti-arthritic properties of cherries [24,27,70]. Further, long term, randomized, double blinded and placebo controlled human trials are needed to confirm anti- arthritic effects of cherry products.

### 2.4. Effects of Consuming Cherries on Sleep, Mood, and Cognitive Functions

Both quality and quantity of sleep were improved by the consumption of sweet [38,53] as well as tart cherries [65,93]. Effect on sleep could be detected within 3 days of consuming sweet cherries (141 g or 25 cherries/day) and within 5 d of consuming tart cherries (240 mL of tart cherry juice; approximately 100 cherries/day). The studies using sweet cherries also reported a decrease in urinary cortisol and anxiety, and improved mood [38,94]. Those functions were not tested in the studies using tart cherries [65,93]. However, mood and cognitive functions were not altered within 5 h of supplementing with 60 mL (approximately 180 tart cherries) of tart cherry concentrate [61]. Similarly, there was no significant difference in cognitive functions tested at 0 and 6 h after a single serving of cherry juice (300 mL, anthocyanins 55 mg) to young and older adults [95]. Authors suggested that the lack of an effect may be due to the low dose of anthocyanins served. While there are only limited numbers of published studies testing the effects of cherries on cognitive functions, several studies assessed the effects of other anthocyanin rich foods on cognitive functions. Thus, cognitive functions were improved in 6 out of 7 human intervention studies using food-based anthocyanins [62]. Similarly, 17 out of 19 epidemiological studies reported significant benefits of fruit, vegetable, or juice consumption on cognitive functions [96].

Serum cortisol levels did not differ between placebo and tart cherry groups (100–120 cherries/day, 5 days before and on the day of race) in marathon runners before and end of race; or 24 and 48 h after the race [50]. In another study, which involved weight lifting the serum cortisol at 60 min post exercise was significantly greater in the cherry consuming group compared with the placebo [46]. Yet, in another marathon race study by the same authors, the serum cortisol at 60 min after the race was significantly lower in the cherry group compared with that in the placebo group [41]. These differences in the cortisol response may be related to the type of exercise, because supplementation with cherries did not alter the serum markers of oxidative stress and inflammation in the study involving weight lifting, while levels of these markers were decreased by consumption of cherries in the marathon runners.

Supplementing diets of aged rats with tart cherry powder improved working memory and autophagy [97], and sweet cherry polyphenols protected cultured neuronal cells from damage by increased oxidative stress [98]. Anthocyanins in animal models improved memory [99,100], and prevented amyloid beta induced Alzheimer disease [101,102]. The results from these animal and cell culture studies are suggestive of improved cognitive function in humans consuming cherries. Overall, these reports support further examination of the possible cognitive enhancing effects of cherry consumption.

## 3. Conclusions

Evidence from published reports is reasonably strong to indicate that consumption of cherries decreased markers for oxidative stress, inflammation, exercise-induced muscle soreness and loss of strength, and blood pressure acutely after ingesting cherries. Limited numbers of published reports also indicate beneficial effects of consuming cherries on arthritis, diabetes, blood lipids, sleep, cognitive functions, and possibly mood. It should be noted that many of these studies, which suggested health benefits of cherry consumption, used amounts (45–270 cherries/day) that might be considered to be a high dose. Because of the finite number of studies and some inconsistencies among the results, additional studies are needed to support these claims. Several factors, including number of study participants and their health status, composition of basal diet, duration of supplements, anthocyanin concentration and composition, compliance, sensitivity, and precision of the analytical methods may have contributed to the discrepancies among the published reports. Developments of stable and standardized cherry products that retain nutrient composition of fresh cherries and of placebos devoid of polyphenols are desperately needed to precisely assess the health promoting effects of cherry consumption. It is important that all intervention studies report at least the daily total amounts of phenolics and anthocyanins served to study participants. Additional studies are also needed to understand the underlying mechanisms that may confer health benefits of cherry consumption.

## Figures and Tables

**Table 1:** List of Cherry studies investigating biological or clinical markers for pre-disease and disease conditions.

Medical Condition	Investigators Who Examined the Effect of Cherries or Cherry Products on Markers for Listed Conditions
Oxidative stress	Total studies 10. ↓ in 8 studies [37,38,39,40,41,42,43,44,45]. No change in 2 studies [46,47].
Inflammation	Total studies 16. ↓ in 11 studies [37,38,39,40,41,44,46,48,49,50,51,52]. ↑ in 1 study [53]. No change in 4 studies [47,54,55,56].
Exercise induced pain, muscle damage, and recovery	Total studies 9. ↓ Pain, soreness, or muscle damage in 8 studies [37,39,41,44,46,49,57,58]. No change in 1 study [47].
Risk factors for diabetes and cardiovascular disease	↓ HbA1C in diabetic women [59]; no change in fasting glucose or insulin [23,45,51] in healthy subjects; ↓VLDL & TG/HDL ratio in obese [52] but no change in VLDL, LDL, HDL, TG, lipoprotein particle size and number in healthy [23,42,45]. ↓ SBP [51,54,60,61]; ↓ both SBP and DBP [59,62]; No change in either SBP or DBP [42]. ↓ ENRAGE, EN-1, PAI-1 [51]
Arthritis and associated risk factors	↓ gout attacks [63,64]; ↓ Osteoarthritis [55]; ↓ plasma uric acid [40,52,63]
Sleep	Total 4 studies. ↑ quantity and quality of sleep [38,39,53,65,66]
Stress, anxiety, mood, memory and cognitive functions	↓ Urinary cortisol, stress, anxiety, and improved memory, mood, and cognitive functions [19,38]. NC in cognitive functions within 5 h of a TC concentrate [60]. Serum cortisol ↓ [41,46] and NC [50].

VLDL, very low density lipoprotein; TG/HDL, triglycerides/high-density lipoprotein; HDL, high-density lipoprotein; LDL, low-density lipoprotein; SBP, systolic blood pressure; DBP, diastolic blood pressure; ET-1, endothelin-1; ENRAGE, extracellular newly identified ligand for the receptor for advanced glycation end products; PAI-1, plasminogen activator inhibitor-1; NC, no change; TC, tart cherry.

**Table 2:** Effects of cherries and products made from cherries on biological and clinical markers of human health.

Reference	Study Subjects	Study Design	Treatment	Major Findings	Comments
**Oxidative Stress**					
[37]	10 well trained male athletes (27.8 ± 1.6 y., Mean ± SD)	CO, 7 d prior, 1 d of single leg extensions and 2 d post exercise; W/O 2 wk.	30 mL TCJ or placebo (isoenergetic fruit concentrate) b.i.d.	Recovery of maximum voluntary contractions faster after TCJ than placebo.	No effect of TCJ on serum CRP, nitrotyrosine and CK.
[38,66]	Young, middle aged and elderly (3 M + 3 F in each group, 20–30, 45–55, 65–75 y.),	Before and after treatment, 3 d each.	3 d basal level and 3 d SC powder. (141 g cherries/serving) b.i.d.	Total sleeping time, immobility, and antioxidant capacity SC powder > basal level. Sleep latency SC < basal.	SC powder improved sleep and antioxidant status in all age groups.
[40]	10 healthy women, 22–40 y.	Blood and urine collected at 0, 1.5, 3 and 5 h after treatment.	Single bolus of Bing sweet cherries (SC), (280 g).	↓ in plasma ORAC and FRAP, and ↑ in urinary UA at 1.5, 3 and 5 h; ↓ in plasma UA at 5 h.	SC intake ↓ plasma oxidative stress and UA.
[41]	27 endurance trained runners or triathletes (21.8 ± 3.9 y, Mean ± SD)	Parallel, PC, 10 d. Blood samples taken pre, 60 min, 24 and 48 h post exercise.	Same supplements and protocol as above. TC *n* = 11 and placebo *n* = 18.	TC improved marathon time and ↓ markers of muscle catabolism (creatinine, total protein and cortisol) oxidative stress and inflammation when compared with placebo.	TC supplements may improve recovery from exercise-induced stress.
[42]	47 healthy adults (30–50 y.)	Randomized, parallel, PC, 6 wk.	30 mL TC concentrate (anthocyanins 270 mg/d) or placebo.	↑ FRAP, but no difference in SBP, DBP, CRP, total- and HDL-C.	Lack of an effect on BP may be due to low dose of anthocyanins and healthy participants.
[43]	6 M + 6 F (61–75 y.)	Randomized, CO, PC, 2 wk. each treatment, W/O 4 wk.	240 mL TCJ or placebo (Kool Aid) b.i.d.	TCJ ↓ plasma F2-isoprostane and urinary 8-hydroxyguanosine, placebo had no effect.	TCJ ↓oxidative stress in elderly.
[46]	23 resistance trained men (20.9 ± 2.6 y., Mean ± SD)	Randomized, parallel, PC, 10 d. Blood samples taken pre, 60 min., 24 and 48 h post exercise.	TC (*n* = 11) or placebo (*n* = 12) powder (480 mg/d) 7 d pre-, 2 d post-and d of exercise. (TC powder approx. equals 300 mL TCJ).	TC ↓ post-exercise muscle soreness. 48 h post-exercise AST, ALT and creatinine ↓ by TC compared with pre-. No change in serum markers of oxidative stress and inflammation.	TC improved recovery and muscle soreness but not markers of oxidative stress and inflammation.
[47]	9 highly-trained male Water polo players (18.6 ± 1.4 y., Mean ± SD)	Randomized, CO, PC, each period 7 d. W/O 5 wk. Blood drawn d 1 before supplement, d 6 pre- and post exercise; d 7 pre-exercise.	30 mL TCJ or placebo in a.m. and 60 mL p.m. after exercise on d 1–7 (total equivalent to 270 TC/d).	D 6 post exercise IL-6 TCJ > placebo. CRP, UA, F2 isoprostane on all test days and IL-6 on d 1 and 7 did not differ between TCJ and placebo. No difference in measures of performance and recovery.	Non-weight bearing sports may not have caused substantial oxidative stress and inflammation to observe any benefits of TCJ.
[44]	16 trained cyclists (30 ± 8 y., Mean ± SD)	Randomized, CO, PC, TCJ or placebo 8 d; W/O 14 d. Stochastic cycling on d 5, 6, 7.	30 mL TCJ conc. or placebo (Kool Aid) at 8 a.m. and 6 p.m. (approx. 200 TC/d).	Serum CRP, IL-6, and lipid hydroperoxides TCJ < placebo in blood samples taken on post-trial d 5, 6 and 7.	TCJ ↓ cycling induced CRP, IL-6 and lipid peroxidation.
[45]	Same as in reference #19	FBG and urinary anti-oxidant capacity measured, before, 5 d after, and 1 d post SC supplement.	Same as in reference #19.	No difference in FBG, but urinary antioxidant capacity ↑ when compared to placebo.	Since anthocyanins improve insulin secretion, it is possible that SC may ↓ FBG if monitored within 2 h of their intake.
**Inflammation**					
[48]	Healthy 11 M + 1F (26 ± 3 y., Mean ± SD,)	Randomized, CO, 2 doses. Blood drawn at 0, 1, 2, 3, 5, 8, 24, 26, and 48 h after TCJ intake. W/O 10 d.	30 or 60 mL TCJ (apporx.100 or 200 TC).	Serum CRP and UA ↓ within 3 h of TCJ intake and remained low until 8 h; Urinary UA ↑ within 3 h and returned to basal level at 8 h	The dose of the TCJ had no effect, suggesting 30 mL TCJ was adequate to provide maximum effect.
[37]	10 well trained male athletes (27.8 ± 1.6 y., Mean ± SD)	CO, 7 d prior, 1 d of single leg extensions and 2 d post exercise; W/O 2 wk.	30 mL TCJ or placebo (isoenergetic fruit concentrate) b.i.d.	Recovery of maximum voluntary contractions faster after TCJ than placebo.	No effect of TCJ on serum CRP, nitrotyrosine and CK.
[39]	13 M + 7 F, (37 ± 13 y., Mean ± SD,) marathon athletes	Parallel, PC; TCJ (7M + 3 F), placebo (6 M + 4 F) 5 d before, 1 d during and 2 d post-race.	240 mL TCJ or placebo (Kool Aid) b.i.d. (approx. 100 TC/d).	Exercise associated ↑ in serumCRP, IL-6, muscle damage and pain, Placebo > TCJ. Total serum antioxidant status TCJ > placebo.	TCJ ↓ marathon induced inflammation and pain.
[40]	10 healthy women, 22–40 y.	Blood and urine collected at 0, 1.5, 3 and 5 h after treatment.	Single bolus of Bing sweet cherries (SC), (280 g).	↓ in plasma ORAC and FRAP, and ↑ in urinary UA at 1.5, 3 and 5 h; ↓ in plasma UA at 5 h	SC intake ↓ plasma oxidative stress and UA.
[41]	27 endurance trained runners or triathletes (21.8 ± 3.9 y, Mean ± SD)	Parallel, PC, 10 d. Blood samples taken pre, 60 min., 24 and 48 h post exercise.	Same supplements and protocol as above. TC *n* = 11 and placebo *n* = 18.	TC improved marathon time and ↓ markers of muscle catabolism (creatinine, total protein and cortisol) oxidative stress and inflammation when compared with placebo.	TC supplements may improve recovery from exercise-induced stress.
[46]	23 resistance trained men (20.9 ± 2.6 y., Mean ± SD)	Randomized, parallel, PC, 10 d. Blood samples taken pre, 60 min., 24 and 48 h post exercise.	TC (*n* = 11) or placebo (*n* = 12) powder (480 mg/d) 7 d pre-, 2 d post-and d of exercise. (TC powder approx. equals 300 mL TCJ).	TC ↓ post-exercise muscle soreness. 48 h post-exercise AST, ALT and creatinine ↓ by TC compared with pre-. No change in serum markers of oxidative stress and inflammation.	TC improved recovery and muscle soreness but not markers of oxidative stress and inflammation.
[47]	9 highly-trained male Water polo players (18.6 ± 1.4 y., Mean ± SD)	Randomized, CO, PC, each period 7 d. W/O 5 wk. Blood drawn d 1 before supplement, d 6 pre- and post exercise; d 7 pre-exercise.	30 mL TCJ or placebo in a.m. and 60 mL p.m. after exercise on d 1–7 (total equivalent to 270 TC/d).	D 6 post exercise IL-6 TCJ > placebo. CRP, UA, F2 isoprostane on all test days and IL-6 on d 1 and 7 did not differ between TCJ and placebo. No difference in measures of performance and recovery.	Non-weight bearing sports may not have caused substantial oxidative stress and inflammation to observe any benefits of TCJ.
[44]	16 trained cyclists (30 ± 8 y., Mean ± SD)	Randomized, CO, PC, TCJ or placebo 8 d; W/O 14 d. Stochastic cycling on d 5, 6, 7.	30 mL TCJ conc. or placebo (Kool Aid) at 8 a.m. and 6 p.m. (approx. 200 TC/d).	Serum CRP, IL-6, and lipid hydroperoxides TCJ < placebo in blood samples taken on post-trial d 5, 6, and 7.	TCJ ↓ cycling induced CRP, IL-6 and lipid peroxidation.
[49]	16 healthy male soccer players	Randomized, CO, PC, TCJ or placebo 8 d; baseline, 24, 48, 72 h post exercise.	30 mL TCJ conc. or placebo (Kool Aid) twice a day.	TCJ improved performance, recovery and muscle soreness, and ↓ serum IL-6.	No effect of TCJ on LOOH and CK, and CRP.
[50]	20 marathon runners	Randomized, TCJ (7 M + 3 F) or placebo (6 M + 4 F) 5 d before, 1 d during and 2 d post-race.	TCJ or placebo as listed in 41.	Incidence and severity of URTS and ↑ in plasma CRP at 24 and 48 post race was greater in placebo than TCJ.	TCJ ↓ post-marathon development of URTS.
[51]	2 M + 16 F, 45–61 y., BMI 20–30 kg/m^2^, mild ↑ in CRP	CO with blood drawn at −7, 0, 14 and 28 d of SC intake; also 28 d after discontinuation.	280 g depitted SC/d (45 SC) replacing dietary carbohydrates.	SC ↓ plasma conc. of CRP, IL-18, ENRAGE, PAI-1, ET-1, TNF α, EGF, ferritin, RANTES, NO and ↑ IL-1Ra.	SC intake ↓ plasma markers of CVD, arthritis, hypertension, diabetes, cancer and inflammation.
[52]	10 over weight and obese, (38.1 ± 12.5 y., BMI 32.2 ± 4.6).	Randomized, CO, TCJ or placebo beverage 4 wk.; W/O 2 wk.	240 mL TCJ or placebo beverage/d.	TCJ ↓ serum UA, TNF α, MCP-1, ESR, TG, and VLDL compared with placebo.	TCJ ↓ inflammation and risk factors for gout and CVD.
[54]	49 subjs over the age of 70 with dementia	Randomized, parallel, PC, *n* = 24 in SC, and 25 in placebo groups.	200 mL Bing SC or apple juice once a day for 12 wk. Responses tested at 6 and 12 wk.	SCJ improved verbal fluency, short term memory and ↓ SBP both at 6 and 12 weeks. No change in fasting serum IL-6 and CRP.	200 mL of SCJ provided 138 mg anthocyanins/d, which may not be enough to ↓ inflammation.
[55]	44 M + 14 F (56.7 ± 11.3 y. non-diabetic grade 2–3 OA patients	Randomized, CO, PC, TCJ or placebo 6 wk.; W/O 1 wk.	240 mL TCJ or placebo (Kool Aid) b.i.d.) (approx. 100 TC/d).	TCJ ↓ arthritis index, pain, stiffness and function compared with placebo.	No change in serum CRP.
[56]	Overweight and obese 37 men (61.4 ± 7.7 y., BMI 31.7 ± 4.3)	Before and after SC consumption; no control group.	142 g fresh SC 3 times a day, 4 wk.	Urinary PGEM, TBX2, serum CRP and homocysteine did not change with SC consumption.	Anthocyanin content of the different batches of SC used varied several folds.
[53]	Same as in reference #19	Same as in reference #19.	Same as in reference #19.	SC ↓ sleep latency, number of awakenings, ↑ sleep time and immobility.	↑ IL-1 β, IL-8, TNF α in blood drawn at 1 a.m.; perhaps caused by 5-hydroxyindocle acetic acid.
**Exercised Induced Pain, Muscle Damage and Recovery**					
[37]	10 well trained male athletes (27.8 ± 1.6 y., Mean ± SD)	CO, 7 d prior, 1 d of single leg extensions and 2 d post exercise; W/O 2 wk.	30 mL TCJ or placebo (isoenergetic fruit concentrate) b.i.d.	Recovery of maximum voluntary contractions faster after TCJ than placebo.	No effect of TCJ on serum CRP, nitrotyrosine and CK.
[39]	13 M + 7 F, (37 ± 13 y., Mean ± SD,) marathon athletes	Parallel, PC; TCJ (7M + 3 F), placebo (6 M + 4 F) 5 d before, 1 d during and 2 d post-race.	240 mL TCJ or placebo (Kool Aid) b.i.d. (approx. 100 TC/d).	Exercise associated ↑ in serum CRP, IL-6, muscle damage and pain, Placebo > TCJ. Total serum antioxidant status TCJ > placebo.	TCJ ↓ marathon induced inflammation and pain.
[41]	27 endurance trained runners or triathletes (21.8 ± 3.9 y., Mean ± SD)	Parallel, PC, 10 d. Blood samples taken pre, 60 min., 24 and 48 h post exercise.	Same supplements and protocol as above. TC *n* = 11 and placebo *n* = 18	TC improved marathon time and ↓ markers of muscle catabolism (creatinine, total protein and cortisol) oxidative stress and inflammation when compared with placebo.	TC supplements may improve recovery from exercise-induced stress.
[46]	23 resistance trained men (20.9 ± 2.6 y., Mean ± SD)	Randomized, parallel, PC, 10 d. Blood samples taken pre, 60 min., 24 and 48 h post exercise.	TC (*n* = 11) or placebo (*n* = 12) powder (480 mg/d) 7 d pre-, 2 d post-and d of exercise. (TC powder approx. equals 300 mL TCJ).	TC ↓ post-exercise muscle soreness. 48 h post-exercise AST, ALT and creatinine ↓ by TC compared with pre-. No change in serum markers of oxidative stress and inflammation.	TC improved recovery and muscle soreness but not markers of oxidative stress and inflammation.
[47]	9 highly-trained male Water polo players (18.6 ± 1.4 y., Mean ± SD)	Randomized, CO, PC, each period 7 d. W/O 5 wk. Blood drawn d 1 before supplement, d 6 pre- and post exercise; d 7 pre-exercise.	30 mL TCJ or placebo in a.m. and 60 mL p.m. after exercise on d 1–7 (total equivalent to 270 TC /d).	D 6 post exercise IL-6 TCJ > placebo. CRP, UA, F2 isoprostane on all test days and IL-6 on d 1 and 7 did not differ between TCJ and placebo. No difference in measures of performance and recovery.	Non-weight bearing sports may not have caused substantial oxidative stress and inflammation to observe any benefits of TCJ.
[49]	16 healthy male soccer players	Randomized, CO, PC, TCJ or placebo 8 d; baseline, 24, 48, 72 h post exercise.	30 mL TCJ conc. or placebo (Kool Aid) twice a day.	TCJ improved performance, recovery and muscle soreness, and ↓ serum IL-6.	No effect of TCJ on LOOH and CK, and CRP.
[57]	14 male college students	Randomized, CO, PC 2 wk. W/O; arm eccentric exercise on d 4 of each period.	360 mL TCJ or placebo, b.i.d. for 4 d; each serving equals 50–60 TC.	Exercise associated loss of strength, muscle damage and pain TCJ < placebo.	Placebo used was Kraft Foods, cherry flavored Kool Aid.
[58]	36 M + 18 F (35.8 ± 9.6 y., Mean ± SD), healthy runners	Randomized, parallel, PC; ran 26.3 ± 2.5 km in 24 h TCJ or placebo 7 d prior and on d of race.	TCJ 355 mL b.i.d (19 M and 7F) or placebo (15 M and 10F). About 200 TC/d.	Post run pain score, TCJ 12 ± 18, and placebo 37 ± 20 mm.	TCJ prior to the race ↓ post-race pain.
**Diabetes and Cardiovascular Disease**					
[23]	2 M + 16 F, 45–61 y., BMI 20–30 kg/m^2^, mild ↑ in CRP	CO with blood drawn at −7, 0, 14 and 28 d of SC intake; also 28 d after discontinuation.	280 g depitted SC/d (45 SC) replacing dietary carbohydrates.	SC ↓ plasma conc. of CRP, IL-18, ENRAGE, PAI-1, ET-1, TNF α, EGF, ferritin, RANTES, NO and ↑ IL-1Ra.	SC intake ↓ plasma markers of CVD, arthritis, hypertension, diabetes, cancer and inflammation.
[42]	47 healthy adults (30–50 y.)	Randomized, parallel, PC, 6 wk.	30 mL TC concentrate (anthocyanins 270 mg/d) or placebo.	↑ FRAP, but no difference in SBP, DBP, CRP, total- and HDL-C.	Lack of an effect on BP may be due to low dose of anthocyanins and healthy participants.
[51]	2 M + 16 F, 45–61 y., BMI 20–30 kg/m^2^, mild ↑ in CRP	CO with blood drawn a −7, 0, 14 and 28 d of SC intake; also 28 d after discontinuation.	280 g depitted SC/d (45 SC) replacing dietary carbohydrates.	SC ↓ plasma conc. of CRP, IL-18, ENRAGE, PAI-1, ET-1, TNF α, EGF, ferritin, RANTES, NO and ↑ IL-1Ra.	SC intake ↓ plasma markers of CVD, arthritis, hypertension, diabetes, cancer and inflammation.
[52]	10 over weight and obese, (38.1 ± 12.5 y., BMI 32.2 ± 4.6)	Randomized, CO, TCJ or placebo beverage 4 wk.; W/O 2 wk.	240 mL TCJ or placebo beverage/d	TCJ ↓ serum UA, TNF α, MCP-1, ESR, TG, and VLDL compared with placebo	TCJ ↓ inflammation and risk factors for gout and CVD
[54]	49 subjs over the age of 70 with dementia	Randomized, parallel, PC, *n* = 24 in SC, and 25 in placebo groups.	200 mL Bing SC or apple juice once a day for 12 wk. Responses tested at 6 and 12 wk.	SCJ improved verbal fluency, short term memory and ↓ SBP both at 6 and 12 weeks. No change in fasting serum IL-6 and CRP.	200 mL of SCJ provided 138 mg anthocyanins/d, which may not be enough to ↓ inflammation.
[45]	Same as in reference #19	FBG and urinary anti-oxidant capacity measured, before, 5 d after, and 1 d post SC supplement.	Same as in reference #19.	No difference in FBG, but urinary antioxidant capacity ↑ when compared to placebo.	Since anthocyanins improve insulin secretion, it is possible that SC may ↓ FBG if monitored within 2 h of their intake.
[59]	19 diabetic women, BMI 29.6 ± 4.3	Before and after treatment, 6 wk.	40 g TC concentrate/d (anthocyanins 720 mg/d).	↓ HbA1C, SBP, DBP, total- and LDL-C.	No Control group.
[60]	15 M with early hypertension, SBP > 130, DBP > 80	Randomized, CO, PC, W/O 14 d. Responses tested at 0, 1, 2, 3, 5, and 8 hr) after TC or placebo intake.	60 mL TC concentrate (180 TC) or placebo (fruit flavored cordial).	SBP, TCJ < placebo at 1, 2 and 3 h, with peak reduction at 2 h.	↓ in SBP associated with ↑ in circulating protocatechuic and vanillic acids
[62]	Pilot study with 6 young and 7 older adults	Before and after SCJ consumption; no control group	SCJ served either 300 mL at 0 h or 100 mL at 0, 1, and 2 h; BP monitored at 0, 2 and 6 h	Both SBP and DBP significantly ↓ at 2 h with a single dose but not with split dose; no effect at 6 h	Certain minimum blood concentration of polyphenols is needed to lower BP.
**Arthritis and Associated Risk Factors**					
[40]	10 healthy women, 22–40 y.	Blood and urine collected at 0, 1.5, 3 and 5 h after treatment.	Single bolus of Bing sweet cherries (SC), (280 g).	↓ in plasma ORAC and FRAP, and ↑ in urinary UA at 1.5, 3 and 5 h; ↓ in plasma UA at 5 h	SC intake ↓ plasma oxidative stress and UA.
[52]	10 over weight and obese, (38.1 ± 12.5 y., BMI 32.2 ± 4.6)	Randomized, CO, TCJ or placebo beverage 4 wk.; W/O 2 wk.	240 mL TCJ or placebo beverage/d.	TCJ ↓ serum UA, TNF α, MCP-1, ESR, TG, and VLDL compared with placebo.	TCJ ↓ inflammation and risk factors for gout and CVD.
[55]	44 M + 14 F (56.7 ± 11.3 y. non-diabetic grade 2–3 OA patients	Randomized, CO, PC, TCJ or placebo 6 wk.; W/O 1 wk.	240 mL TCJ or placebo (Kool Aid) b.i.d.) (approx. 100 TC/d).	TCJ ↓ arthritis index, pain, stiffness and function compared with placebo.	No change in serum CRP.
[63]	12 gouty arthritis patients	Before and after treatment, 3 d-3 month.	Fresh or canned tart cherries (TC) 227 g/d.	Blood UA normalized and no attacks of arthritis in all subjs; ↑ freedom of joint use in 4.	↓in Blood UA positively associated with ↓ in gout attacks.
[64]	633 patients with gout	Case-CO, with or without fresh cherries or extract for 2 d prior to gout attack.	Fresh cherries or extract, or without both for 2 d prior to gout attack.	Supplements ↓ gout attacks by 35% compared to control, independent of sex, obesity, alcohol, and drugs.	Attack risk ↓ by 75% when cherry intake was combined with allopurinol use than without either.
**Sleep**					
[38,66]	Young, middle aged and elderly (3 M + 3 F in each group, 20–30 45–55, 65–75 y.),	Before and after treatment, 3 d each	3 d basal level and 3 d SC powder. (141 g cherries/serving) b.i.d.	Total sleeping time, immobility, and antioxidant capacity SC powder > basal level. Sleep latency SC < basal	SC powder improved sleep and antioxidant status in all age groups.
[39]	13 M + 7 F, (37 ± 13 y., Mean ± SD,) marathon athletes	Parallel, PC; TCJ (7M + 3 F), placebo (6 M + 4 F) 5 d before, 1 d during and 2 d post-race	240 mL TCJ or placebo (Kool Aid) b.i.d. (approx. 100 TC/d)	Exercise associated ↑ in serum CRP, IL-6, muscle damage and pain, Placebo > TCJ. Total serum antioxidant status TCJ > placebo.	TCJ ↓ marathon induced inflammation and pain.
[53]	Same as in reference #19	Same as in reference #19	Same as in reference #19	SC ↓ sleep latency, number of awakenings, ↑ sleep time and immobility.	↑ IL-1 β, IL-8, TNF α, in blood drawn at 1 a.m.; perhaps caused by 5-hydroxyindocle acetic acid.
[65]	15 adults, 65 y. or older with chronic insomnia	Randomized, CO, PC, 2 wk. TCJ or placebo each, W/O 2 wk.	240 mL TCJ or placebo (Kool Aid) b.i.d,	TCJ ↓ insomnia severity, but not sleep latency or sleep efficiency	Insomnia and the age of subjects may have lessened the effects of TCJ.
**Stress, Anxiety, Mood, Memory, and Cognitive Function**					
[19]	Young, middle aged and elderly, 5 M and 5 F in each group.	Randomized, CO, PC, W/O 1 wk. Blood and urine collected before, 5 d after and 1 d post supplement.	5 d supplement with dried SC or placebo powder with lunch and dinner (280 fresh cherries SC/d).	SC improved mood, ↓ anxiety and urinary cortisol; ↑ urinary 5-hydroxyindocle acetic acid	SC ↓ stress and anxiety
[38]	Young, middle aged and elderly (3 M + 3 F in each group, 20–30 45–55, 65–75 y.),	Before and after treatment, 3 d each.	3 d basal level and 3 d SC powder. (141 g cherries/serving) b.i.d.	Total sleeping time, immobility, and antioxidant capacity SC powder > basal level. Sleep latency SC < basal.	SC powder improved sleep and antioxidant status in all age groups.
[41]	27 endurance trained runners or triathletes (21.8 ± 3.9 y., Mean ± SD)	Parallel, PC, 10 d. Blood samples taken pre, 60 min., 24 and 48 h post exercise	Same supplements and protocol as above. TC *n* = 11 and placebo *n* = 18	TC improved marathon time and ↓ markers of muscle catabolism (creatinine, total protein and cortisol) oxidative stress and inflammation when compared with placebo.	TC supplements may improve recovery from exercise-induced stress.
[46]	23 resistance trained men (20.9 ± 2.6 y., Mean ± SD)	Randomized, parallel, PC, 10 d. Blood samples taken pre, 60 min., 24 and 48 h post exercise	TC (*n* = 11) or placebo (*n* = 12) powder (480 mg/d) 7 d pre-, 2 d post-and d of exercise. (TC powder approx. equals 300 mL TCJ).	TC ↓ post-exercise muscle soreness. 48 h post-exercise AST, ALT and creatinine ↓ by TC compared with pre-. No change in serum markers of oxidative stress and inflammation.	TC improved recovery and muscle soreness but not markers of oxidative stress and inflammation.
[50]	20 marathon runners	Randomized, TCJ (7M + 3 F) or placebo (6 M + 4 F) 5 d before, 1 d during and 2 d post-race.	TCJ or placebo as listed in 41.	Incidence and severity of URTS and ↑ in plasma CRP at 24 and 48 post race was greater in placebo than TCJ.	TCJ ↓ post-marathon development of URTS.
[60]	20 M + 10 F (45–60 y.) healthy	Randomized, CO, PC, W/O 14 d. Responses tested at 0, 1, 2, 3, and 5 h after TC or placebo intake.	60 mL TC concentrate (180 TC) or placebo (fruit flavored cordial).	SBP, TCJ < placebo at 1, 2 and 3 h, with peak reduction at 1 h No effect on cognitive functions or mood.	SBP but not DBP rapidly responded to TC intake and the ↓ was transient.

ALT, alanine aminotransferase; AST, aspartate amino transferase; b.i.d, two times a day; BMI, body mass index; CO, cross-over; CRP, C-reactive protein; d, day; CVD, cardiovascular disease; CK, Creatinine; DBP, diastolic blood pressure; ET-1, endothelin-1; ENRAGE, extracellular newly identified ligand for the receptor for advanced glycation end products; ESR, erythrocyte sedimentation rate; F, female; FBG, fasting blood glucose; FRAP, ferric reducing ability of plasma; h, hour; IL, interleukin; IL-1Ra, IL-1 receptor antagonist; M, male; min, minute; mo, month; MCP-1, monocyte chemoattractant protein-1; NC, no change; NO, nitric oxide; OA, osteoarthritis; ORAC, oxygen radical absorbing capacity; PAI-1, plasminogen activator inhibitor-1; PC, placebo controlled; PGEM, prostaglandin E2 metabolite; RANTES, regulated upon activation, normal T cell expressed and secreted; SBP, systolic blood pressure; SC, sweet cherry; SCJ, sweet cherry juice; TC, tart cherry; TCJ, tart cherry juice; TBX2, thromboxane B2; TG, triglyceride; TNF α, tumor necrosis factor alpha; UA, uric acid; URTS, upper respiratory tract symptoms; VLDL, very low density lipoprotein; W/O, wash out; wk., week; y., years; LDL, low-density lipoprotein; HDL, high-density lipoprotein.
